# Crop-livestock integration practices, knowledge, and attitudes among smallholder farmers: Hedging against climate change-induced shocks in semi-arid Zimbabwe

**DOI:** 10.1515/biol-2021-0135

**Published:** 2021-12-31

**Authors:** Joseph P. Musara, Handsen Tibugari, Busani Moyo, Chinomukutu Mutizira

**Affiliations:** Department of Crop Science, Gwanda State University, P.O. Box 30, Filabusi, Zimbabwe; Department of Animal Science, Gwanda State University, P.O. Box 30, Filabusi, Zimbabwe; Department of Agritex, Insiza District Office, P.O. Box 70, Filabusi, Zimbabwe

**Keywords:** climate change resilience, smallholder farmers, semi-arid area, crop-livestock integration, stakeholder networking

## Abstract

Domestic and international crops and livestock trade remain fragile among Zimbabwean smallholder farmers. Commercial crop-livestock integration in climate change vulnerable areas is low and sparsely documented. Practice, knowledge, and attitude indicators influencing participation of smallholder farmers in crop-livestock integrated platforms as a hedge against climate change-induced risks and uncertainties were assessed. A survey with 240 farmers in Insiza district, Matabeleland province, Zimbabwe was conducted. A modified knowledge, attitude, and perception framework was used to analyze data from six wards supported by World Vision through supplementary livelihood programs on crop-livestock integration. Conventional crop-livestock (63%), mixed crops-livestock (25%), and traditional grains-livestock (12%) options were dominant. There was a thin presence of stakeholders with a limited number of local buyers, contracting companies, and agro-dealers who participate on these platforms. Farmers have the knowledge, positive attitude, and motivated perceptions about the potential of traditional grains-livestock mechanisms to reduce climate change welfare compromising factors. Unbalanced policies, limited financing, and uncompetitive marketing channels limit the uptake of this option. Traditional grains-livestock alternatives should be supported in semi-arid environments to reduce food, income, and nutrition insecurity. Public-private partnerships should establish value addition systems to increase the market size of traditional grains-livestock products and enhance commercialization.

## 1 Introduction

### 1.1 Background and context

Agriculture is an important sector for sustaining the development prospects of Zimbabwe’s economy [1]. This article focuses on smallholder farmers who mainly produce food crops on small pieces of land with limited scope for cash crops and are essential players in contributing towards the national agricultural output. In the 1990s, they contributed about 70% of the total national throughput [2]. However, those domiciled in semi-arid areas of Zimbabwe are currently playing second fiddle in terms of crop and livestock production and marketing activities. They typically have limited access to key resources such as productive land, capital (both physical and financial), knowledge, food production techniques, and markets. This is compromised by their use of rudimentary production technologies and the low adoption of emerging innovations. The matrix of challenges creates asymmetries that compromise their effective integration in strategic agricultural value chain platforms [3] to enjoy competitive advantages such as crop-livestock integrated systems. The risky and changing climatic conditions in which these smallholder farmers operate further compound this scenario and affect the decisions made across “competing” crop and livestock enterprises [4]. This manifests especially when the primary factors such as land and water are also significantly limiting [5]. In the semi-arid areas, a monoculture agriculture game-plan, which over supports maize across geospatial areas greatly exposes the smallholder farmers to unpredictable markets, biased support programs, and climate change [6]. Therefore, there is an urgent need to design and sustain strategies that create the diversification scope with crops and livestock within an integrated framework to reduce the extent of risk exposure for small-scale farmers [7,8]. However, currently, on the one hand, there is evidence that the main hurdle for cereal crop production system is the unsustainably low market price [9]. On the other hand, livestock production is affected by persistent disease outbreaks and low water levels at drinking points during lean times of the season, especially in the semi-arid areas. This is also compounded by the weak marketing arrangements, which offer unrewarding returns to small-scale livestock producers who are left at the mercy of unethical middlemen.

The semi-arid areas of Zimbabwe are characterized by low, often erratic, and unreliable rainfall coupled with extremely high temperatures. This makes dryland crop and livestock farming in these areas almost impossible. There is evidence that most rural farming communities in semi-arid areas do not have the resources to militate against changes in their natural, social, institutional, and economic environments. Therefore, they need to adapt to these conditions by changing their cropping and livestock systems as guided by government policy, e.g., subsidies [10]. The idea is to adopt those systems that are resistant and tolerant to the unfavorable agricultural conditions brought about by shocks in their environments [11]. This implies a shift in their crop and livestock enterprise portfolios, which may negatively affect household welfare if the farm enterprises are not in line with their sustainable livelihood requirements. Recently, climate change continues to threaten to erode human freedoms and limit their choices. There are several strategies that farmers can adopt to face climate change, contrasting vulnerabilities, and insecurities. Some of these strategies have been scientifically proven to work well by many research institutes such as CIMMYT [12,13]. Among the most notable climate change, adaptation and welfare security-enhancing strategies are the adoption of drought-resistant crops and crop varieties, and the rearing of drought-tolerant livestock types and breeds. The diversification of livelihoods to cushion farmers from welfare changes associated with fluctuations in climate has also been used. The integration of these strategies has not been widely explored, and the current research is advocating for an exploration of farmers’ practices, knowledge, and attitudes towards crop-livestock integrated options. This will enhance coordination and cooperation among value chain stakeholders in a more networked platform as a way forward.

Although many smallholder farming systems in sub-Saharan Africa depend on the interactions of crop and livestock enterprises, research, extension, agricultural innovations, and policies focus on specialized crop and animal production systems [14]. In these specialized systems, output maximization per unit technological input is viewed as the single most important objective. Mixed farming systems are often viewed as inefficient because of the complex management and resources flow among enterprises. High economic return has been the focus of these specialized systems with less regard to other factors such as the possibility of binding networks which go beyond ecological sustainability and economic risk minimization [15]. Vertical integration is frequently viewed as the economically feasible option for a particular specialized system. A number of agricultural innovations and policies targeting these specialized crops or livestock production systems have been instituted, but the nature of crop-livestock integration, and their importance in smallholder farming systems, have rendered them futile. The multiple objectives and constraints at the farm level (household level) necessitate efficient crop-livestock integration for improved farm productivity [13]. Empirical evidence has shown that interacting ecological, social, economic, and political processes influence crop-livestock integration patterns and trajectories over space and time. A growing understanding is emerging that improved crop and livestock integration can contribute to the resilience of agricultural landscapes by more efficiently utilizing natural resources, improving soil structure and productivity, and reducing production and economic risk [16]. While mixed smallholder farmers of sub-Saharan Africa have multiple objectives and strategies, they respond to variability and diversity in environmental and economic conditions through a continuous adaptive change in farming practices. With regard to crop-livestock integration and agricultural intensification, this implies that farmers are constantly making trade-offs between different management options and adjusting their livelihood strategies accordingly. Thus, intensification and improved efficiency of crop-livestock integration requires not only an understanding of the strategies and trade-offs but also how they are influenced by resource endowments, management skills, knowledge, institutions, and market orientation [17,18]. This hypothesis guides the current study.

The argument is that to transform Zimbabwe’s low productive mixed farming into an economically and ecologically sustainable integrated farming system, farmers need relevant scientific information, skills, and competencies, on top of the physical resources. Current options to improve these mixed smallholder farming systems lie in developing viable production technology options for targeted production systems and improving efficiency with existing resources [6]. Moreover, the complexities of these systems necessitate the critical selection of crop-livestock systems that are aptly integrated and intensified to achieve multiple objectives of the farm household. This improved integration process also requires farmers to increase their knowledge, change perceptions, and realign their practices to achieve sustainable intensification on a commonly coordinated platform with other value chains stakeholders. This is on the background that farmers’ response to agricultural innovation is profoundly influenced by the adaptiveness of the technology to the farmers’ multiple goals as mediated by resource endowments. Using ranking techniques, this research aims to identify and recommend viable crop-livestock integrated options that can be adopted for climate change resilience by different household categories, while exploring their knowledge, attitude, and perceptions on crop-livestock integrated systems.

### 1.2 The conceptual framework

**Figure 1:**
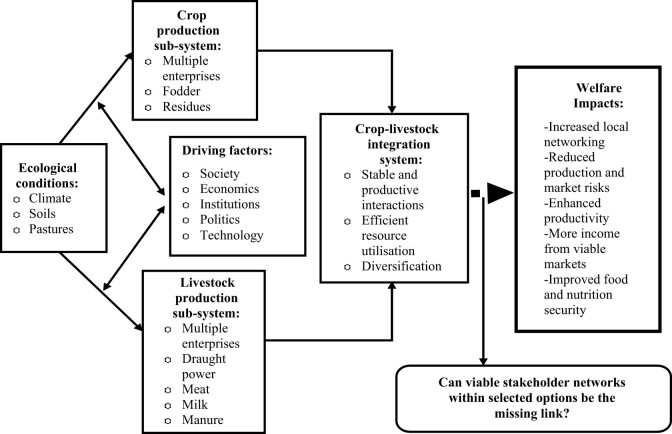
A conceptual perspective of crop-livestock integration networking systems. Adapted from Mkuhlani et al. [12] and Musara et al. [20].

Zimbabwe lags behind a number of southern African countries in research related to crop-livestock integrated systems and their welfare-enhancing potential [12,19]. However, there has been some work done by CIMMYT-Zimbabwe and ICRISAT-Zimbabwe in areas such as Mutoko, Matobo, and Goromonzi concerning the potential of crop-livestock integration systems in the smallholder farming communities. Regardless of these studies, there is still a critical knowledge gap regarding how specific decisions to participate on particular crop-livestock integrated platforms are made in the context of knowledge, attitude, and perception (KAP) frameworks. Therefore, the current study aims to bridge the research gap on sustainable production and marketing decisions in arid and semi-arid areas, which has recently alienated commercial crop-livestock integration more than before. This study postulates that establishing this knowledge and information can act as a strong basis for stakeholders to realign their resources, enhance resource productivity, and help commercialize crop-livestock systems in the study area. As a result, the research was guided by the framework in Figure 1.

The study accepts the existence of a series of possible feeding nodes amongst the strategic climate change mitigation options, and we argue that there can be direct or indirect interconnections among these. Based on experiences reported by, for example, Soussana [10], our visionary hope is that enhancing commercially oriented production of, for example, forage crops by households and their utilization for livestock feeding can increase the herd. Additionally, those who have livestock can improve their reliability in offering draught power services to the community at a reasonable fee, thereby reducing incidences of late land preparation, which is currently common in these localities. Nutritional benefits will also be attained as meat, milk, and their by-products will be readily available at affordable prices in local and external markets.

Indirectly, a market-oriented and integrated crop-livestock production system that harnesses sustainable intensification “ideologies” can offer a breakthrough for smallholder farmers in semi-arid areas. The intuition is to break the problem of inefficient utilization of, for example, manure to improve soil fertility, which is poor in these localities due to limited rotation practices and over-application of conventional remedies such as inorganic fertilizers. This could also have trickling effects on economic and ecological sustainability when manure stock becomes an immediately locally available, relatively cheaper, and sustainable substitute of conventional fertilizers. Inspired by Mkuhlani et al. [12], designing and evaluating these crop-livestock cocktails should help identify appropriate synergies between these two enterprises, which are currently seemingly practiced as mutually exclusive ends. Finally, this might help to provide efficient ecological services. The study isolated provisioning services of increased animal production, supporting services such extension networks, constraints faced by households while on a common networking platform with other supporting stakeholders.

Homann and Van Rooyen [21] observed that it is imperative for game-changing decision-making frameworks in Zimbabwe to explore the social system as an integral aspect in the crop-livestock integration design processes. To achieve this aim, we untangled the various challenges along the selected crop-livestock integrated value chains and identified possible intervention points. We examined the production-market access crossing point and its effect as a possible integral incentive to stakeholder participation decisions on the crop-livestock integrated platforms. We argued that breaking the asymmetry in market information and unlocking potential benefits of networking within and across crop-livestock enterprises should encourage uptake of proposed innovations. The study adopted the “*subsidiarity*” concept of strengthening these integration frameworks. We believe that the impetus to drive the most appropriate crop-livestock integration matrix must be informed by the specific household characteristics, knowledge, and perceptions before considering their position in the broader space of the community and broader value chain. However, we acknowledge that the continuous community interactions in sharing production resources and market information can be useful in reducing the net risks which accrue to the farm-level stakeholders.

## 2 Methodology

### 2.1 Study site description and data collection

The study was performed in the Insiza district of Matabeleland South province. The study site is located at coordinates −20.08422° N, 31.61382° E and lies at an average elevation of 452 m. It is 103 km east of Bulawayo town, along Masvingo-Bulawayo highway, and 105 km southeast of Gwanda town. According to Manatsa et al. [22], the area falls in natural agro-ecological regions IV and V arid and receives an average of 350 mm of erratic and unevenly distributed rainfall per season with temperatures averaging 33°C. The district covers an area of 5,286 square kilometers. It is the third driest district in the province, after Beitbridge and Mangwe districts. This makes the district vulnerable to climate change due to erratic rainfalls and frequent droughts, which consequently lead to food and income insecurity. However, there is a potential to revitalize the livestock value chain, while at the same time producing crops for households and markets’ food needs.

**Figure 2:**
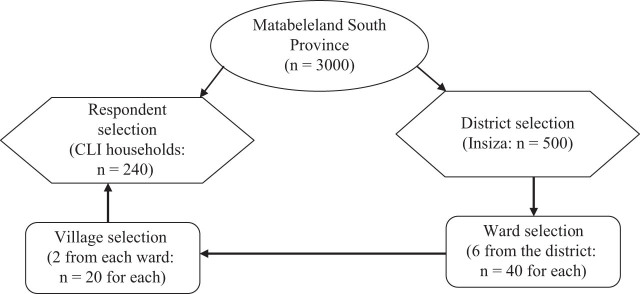
Sampling framework and data collection strategy.

Multistage sampling was used to select the province, district, wards, villages, and households. Figure 2 shows the sampling framework and data collection strategy adopted for the study.

The district was selected because of the high number of active small-scale crop-livestock integration farmers. To account for the geographical and socio-economic variations six wards were randomly selected. Villages and households were then proportionately selected for the study. Informed consent was obtained from all individuals included in this study. The conducted research is not related to either human or animal use. A sample size calculator determined the appropriate number of households to be included in the study. The study adopted a pragmatic philosophy where both qualitative and quantitative methods were used. Creswell [23] postulates that using blended research methods increases the quality of research outcomes. This study then used an exploratory research design to understand relatively unknown and unpredictable smallholder farmers’ views, practices, and attitudes about crop-livestock integrated climate change resilience strategies and their categories in selecting the strategies.

The climate change adaptation strategies were categorized as conventional crop-livestock based, traditional grains-livestock based, and mixed crops-livestock based. Data were collected from the department of agricultural and extension services (Agritex) records at the Insiza district offices. Key informant interviews were also done with Small and Medium Enterprises (SMEs), Insiza Rural District Councilors, Agritex and Veterinary services departments.

### 2.2 Empirical strategy for ranking the climate change adaptation strategies

This study explored and scored the various crop-livestock integration-based climate change adaptation strategies. This was important to get insights into how the specific knowledge, attitudes, and practices are positioned and how they can be re-designed to suit farmer resource endowments and localities. The farmers were asked to prioritize the presented strategies. A Likert scale of 1–5 was then used, and if a response of 1 was recorded, it meant that the farmer viewed the strategy as “least preferred”, while a response of 5 meant the farmer viewed the strategy as “most preferred” [24]. A conversion of the choices was then done into percentage terms using the formula suggested by Farooq et al. [25]:

(1) {S}_{i}=\frac{{X}_{i}}{{\sum }_{i=1}^{n}{X}_{i}},]

where S_*i*_ *=* % score of the *i*th crop-livestock integration climate change resilience strategy; *X*_*i*_ = score of the *i*th crop-livestock integration climate change resilience strategy; ∑*X*_*i*_ = total sum of the score of all the crop-livestock integration climate change resilience strategies; and *i* = 1, 2, 3,…, *n* are the crop-livestock integration climate change resilience strategies.

## 3 Results and discussion

### 3.1 Descriptive statistics of farm-related attributes in the sample

Table 1 summarizes selected production and marketing-related attributes for crops and livestock over the past 5 years. These are critical in explaining the choices made by farmers as they decide whether to integrate the crops and livestock and manage climate change-induced challenges.

**Table 1:** Selected socio-economic characteristics

Parameter	Sampled ward					
	1	11	2	19	9	12
Cattle price/unit (US$)	400	450	400	500	500	450
Goat price/unit (US$)	50	47.5	50	50	40	40
Indigenous chicken price/unit (US$)	5	5	5	5	7	5
Livestock body condition	Poor	Poor	Poor	Poor	Fair	Poor
Pastures availability	Very poor	Very poor	Very poor	Very poor	Poor	Very poor
Livestock poverty death	Low	Low	Low	Low	Low	Low
State of water source (full capacity)	48%	20%	5%	10%	60%	60%
Water for livestock tracking distance (km)	6	7	7	15	5	4
Distance to household water source (km)	3.2	4.5	1.25	7	3	2
Maize price/20 kg tin (US$)	8	6	8	5	7	7
Pearl millet price/20 kg tin (US$)	10	10	10	15	8	10
Rapoko price/20 kg tin (US$)	12	10	12	15	8	10
Sorghum price/20 kg tin (US$)	10	10	10	10	8	10

Source: author analysis.

Table 1 shows the significant variations among the selected wards in terms of the targeted indicators. However, generally, the prices for traditional grains such as sorghum and millets are higher than that for maize. The supply-demand dynamics in the study area showed a mismatch with demand outweighing the supply, thus pushing prices up. This is also in line with Musara et al. [20] findings in a study conducted in the mid-Zambezi valley of Zimbabwe, which noted significantly higher sorghum grain prices relative to maize. Similar findings were also reported by Mukarumbwa and Mushunje [26], who noted the economic viability concerns of sorghum value chain players as the markets determine the prices. Additionally, the scale of sorghum grain production in Zimbabwe pushes the prices up relative to maize which is widely produced at both small and large scales. If adequately supported, this should offer scope for integrating these traditional grains in mainstream decisions for climate change management in the semi-arid areas. Climate change-induced factors such as the availability of pastures and limited water for livestock have also stalled livestock value chain development prospects in the study area. Musemwa et al. [27] also reported similar patterns with small-scale farmers in South Africa in a livestock intensification study.

### 3.2 Crop-livestock integration models used by the smallholder farmers

**Figure 3:**
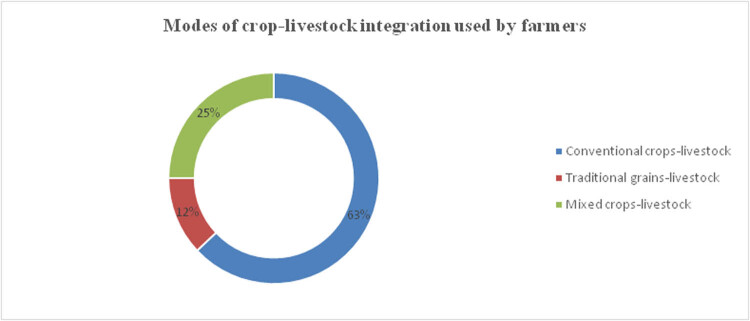
Forms of crop-livestock integration used by the farmers. Source: Author analysis.

Figure 3 shows the different models of crop-livestock integration used by the sampled farmers in the study area.

The most common type of integration is based on conventional crops such as maize and soybeans to meet households’ food and income needs. The farmers using this model accounted for 63% of the total sampled households. They take advantage of the relative convenience associated with these modes of crop and livestock production since the crops are usually supported by government subsidy programs. Only 12% of the sampled farmers have been networked to some contracting companies and use the traditional grains-livestock integration option, as observed in other countries [17]. Key informant interviews showed that these modes are not very common since a few farmers have access to reliable and affordable inputs and markets for traditional grains such as sorghum and millets. The balance still uses the mixed alternative to benefit from subsidies and at the same time also cushion themselves from the unfavorable climatic conditions. Mukarumbwa and Mushunje [26] also made similar observations where farmers spread the climate change-induced risks by producing sorghum as an adaptive crop in the semi-arid areas.

### 3.3 Participating stakeholders on the crop-livestock integration platforms

**Figure 4:**
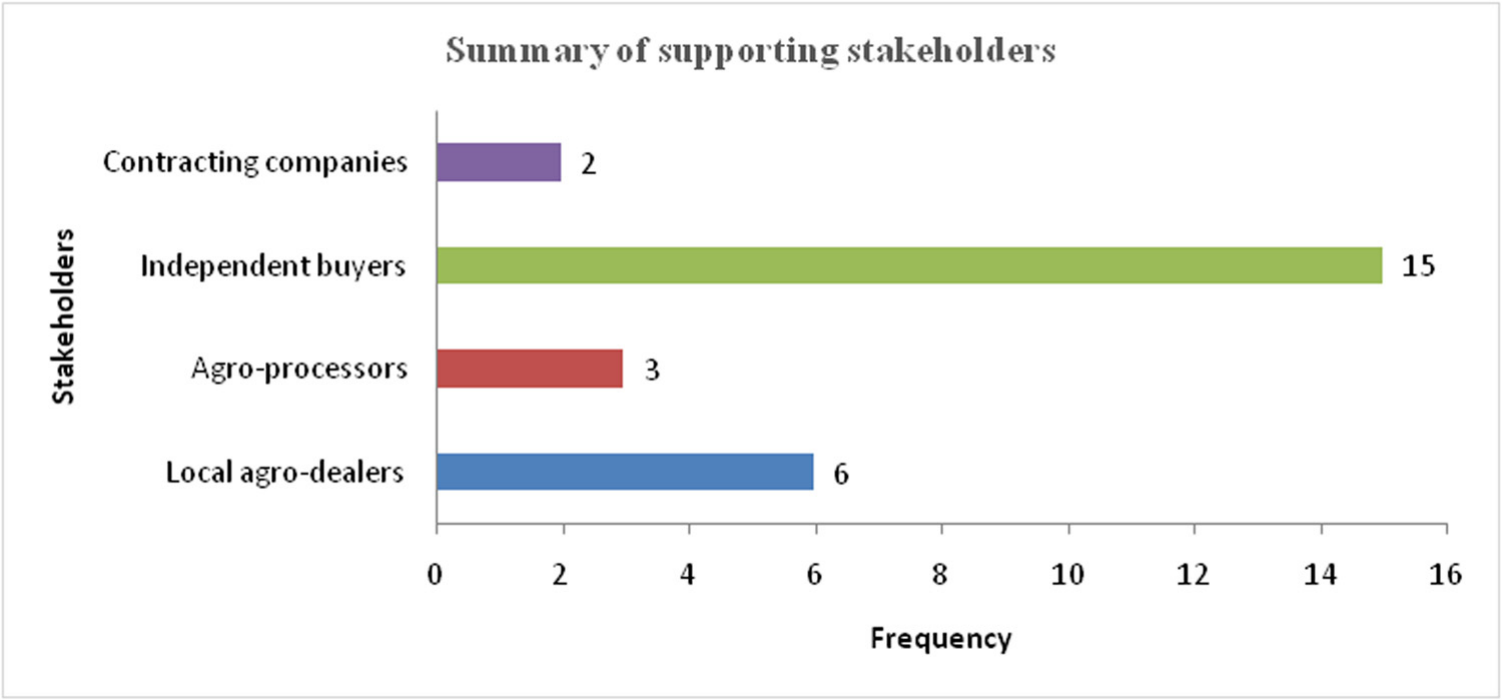
Stakeholders participating on the crop-livestock integration platforms. Source: Author analysis.

Figure 4 shows the stakeholders participating in the various crop-livestock integration activities in the study area.

**Figure 5:**
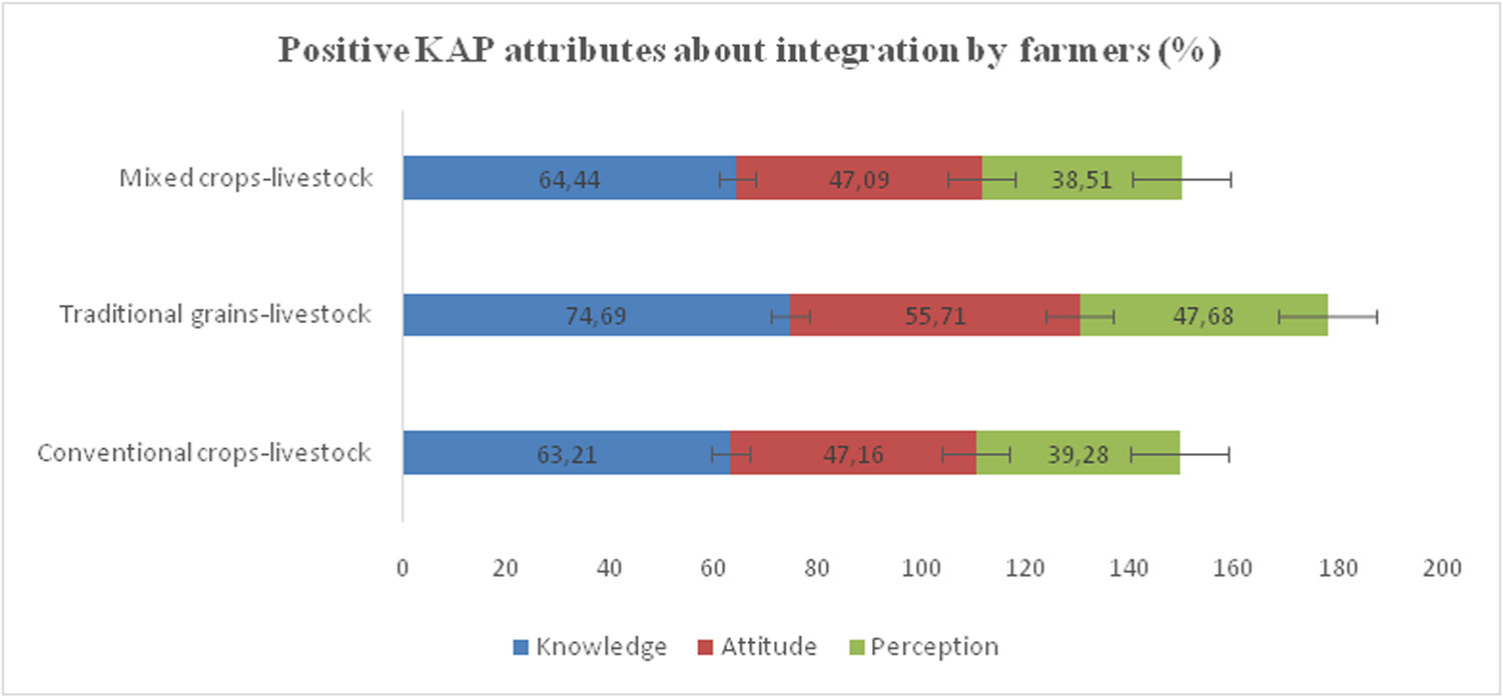
Mean scores for KAP across the crop-livestock integration schemes. Source: Author analysis.

The main stakeholders were the independent buyers, who accounted for 80% of the total sample. These stakeholders have taken business advantage sustained by the willingness of most farmers to make efforts toward increasing productivity for both crops and livestock and target rewarding markets. Local agro-dealers have also emerged strongly and taken up 11% of the market share on the crop-livestock integrated platforms. These have also come on board to bridge the gap left by the mainstream independent buyers who, according to findings from key informant interviews, do not always provide all the required services at the right price and at the right time, thereby compromising the utility of the farmers. The study also looked at the services provided by the contracting companies as other strategic players along the crop-livestock integrated value chain. To explore this further, Figure 5 shows the KAP matrix for the farmers in relation to the applicability of crop-livestock integration modes as climate change management strategies.

The results showed that the traditional grains-livestock schemes scored high across all the three dimensions of the KAP framework. There are indications from the study that this option is more “preferred.” However, due to numerous limitations, farmers do not practice it. This might be due to the motivation to recuperate investments as opposed to the dependency on support from government subsidies, communities, and Non-Governmental Organizations (NGOs). FAO [28] also reviewed time-series evidence on efficiency differences in revolving crop-livestock integrated schemes in a number of countries. Their methodological overview included an indirect measure of farmer participation and resources usage patterns by estimating the frequency of use with particular revolving scheme options. They claimed that this method implies capturing the heterogeneity of variables such as household income and social status, which has a direct bearing on the choice of crop-livestock-based strategies. Asfaw et al. [29] also noted that these individual endowments influence support programs initiated to catalyze participation, especially in rural communities. Thus, it is critical at this stage to note that the structure of the crop-livestock integration strategies and the climate change management services they offer may be complex but necessary to be captured in farmer satisfaction surveys. As mentioned above, a number of many other possible factors may lead to farmer strategies usage differences among various crop-livestock integrated alternatives, and these are also shown in this study.

**Figure 6:**
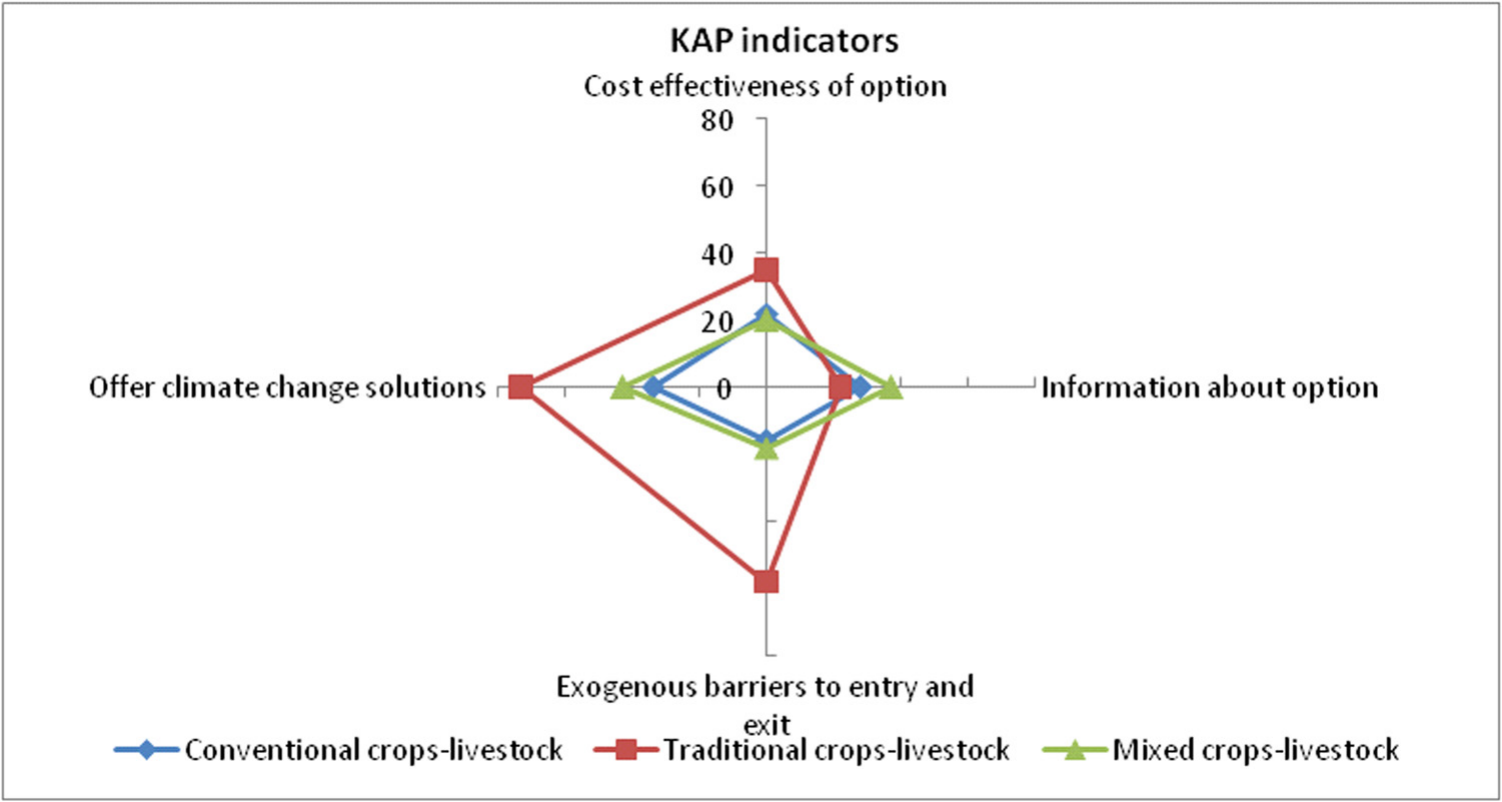
Least square means of KAP scores for the crop-livestock integration schemes. Source: Author analysis.

Comprehensive stakeholder scrutiny was done to understand how stakeholders perceived the various crop-livestock integration choices in terms of their potential to enhance value chains and sustain livelihoods for unlocking community development pathways. A summary of the specific stakeholders’ thematic reactions based on their KAP is presented in Figure 6. Generally, stakeholders reported that the crop-livestock integration options are limited in terms of their usability in the current contexts and hence the low flexibility in the choices across sub-systems of these merged value chains.

Figure 6 also shows that the traditional grains-livestock still emerged as the dominant integration system in terms of ability to offer climate change solutions, cost-effectiveness, and minimal barriers to entry and exit. This creates opportunities for stakeholders to use this option due to its multiple benefits. The mixed crops-livestock option also dominated in terms of the availability of information about production and marketing alternatives along with the value chain nodes. This also provided scope for its adoption by farmers and other stakeholders alike. These observations motivated our further investigation of the cluster-specific constraints limiting the uptake of the various crop-livestock integration alternatives for climate change management. The challenges faced by the farmers in adopting the various crop-livestock integration modes for managing climate change-induced shocks were scored and are shown in Table 2.

**Table 2:** Summary of challenges experienced by the farmers (percent score)

Constraints in using option	Crop-livestock integration mode			
	Conventional crop-livestock	Traditional grains-livestock	Mixed crops-livestock	Total sample
Ineffective extension services	5.34	5.88	5.22	5.66
Unreliable product markets	5.25	5.79	5.28	5.43
Lack of information	4.09	4.56	4.17	4.48
Insufficient finances	4.99	5.12	5.01	5.00
High prices of inputs	4.83	5.09	4.92	5.02
Frequent disease outbreaks	4.47	4.12	4.23	4.31
Low producer prices	4.43	4.45	4.44	4.43
Late delivery of inputs	4.21	4.78	4.53	4.69
Unavailability of inputs	3.99	5.43	4.39	4.64
Labor shortages	3.48	3.21	3.49	3.42

Source: author analysis.

#### 3.3.1 Reducing productive costs using the integrated scheme

About four-fifths of the participating stakeholders expressed the expected benefits of lowered production costs as a result of using efficient crop-livestock integrated schemes. They also noted that for the benefits to be sustainable, there is a need to strengthen communication and other soft skills such as negotiating and bargaining competencies through training support among farmers and independent buyers. This, according to Kreitler et al. [30], is critical to enhance their understanding of the various dimensions of these integrated crop-livestock innovations and how they can play a part in supporting the functions along various value chain pillars. Mkuhlani et al. [12] also reported the need for human capital development programs which can unlock the entrepreneurship capacity of small-scale farmers.

One farmer said:

*“It [the conventional-livestock integrated scheme option] reduces the time of scouting for input and output markets since in most cases the farmers are located closer to their points of marketing. This reduces the transaction costs of doing business and reduces risks. Additionally, most of the inputs for conventional crops such as maize are highly subsidized by the government and NGOs.”*

These findings are also confirmed in empirical work on multiple challenges with maize and sorghum support programs in South Africa by Poonyth et al. [31].

#### 3.3.2 Limited scaling up prospects for the available schemes

Results from Table 2 showed a widely held consensus that stakeholders perceive crop-livestock integration financing program alternatives as having scope for being scaled up by current users and absorbing new users. Financing models, especially for the traditional grains-livestock integrated option, were weakly coordinated. The use of initiatives such as social groups as a platform for the exchange of information among the various farmer support program participants was identified as important by the stakeholders. In agreement with Soussana [10] findings, with small-scale farmers, there is no well-designed platform where stakeholders share direct information on the latest trends in managing climate-smart schemes.

One specialist in crop-livestock integration development management indicated that:

*“Taking advantage of the business potential from scaling up capacity of the various crop-livestock integration scheme platforms, one can be able to make appropriate decisions on how the demand and supply patterns of the markets are moving over time depending on broader socio-economic trends. This determines when, how, and of how frequently the customers will expect to use the scheme goods and services. This is of economic importance because it saves time and other resources within the blended value chain structures*.”

Using the case of gluten-free dough and bread, Cappelli et al. [18] also reported that the emergence of new innovations triggers a sequence of events that need to be supported by sustainable strategies. This, according to Musara et al. [20], can also enhance the scope for scaling up opportunities for value chain functions, especially in climate change-sensitive environments.

#### 3.3.3 Barriers to entry and exit with most schemes

There is evidence from the key informant interviews done during the study that the benefits of those who advocate for uptake of the crop-livestock integration schemes can only be recognized by the informed stakeholders. Homann and Van Rooyen [21] postulate that there are some barriers to effective and sustainable integration of the schemes’ activities in the overall crops and livestock sectors’ performance matrix through unlocking various socio-institutional dimensions. Cappelli and Cini [8] also noted similar patterns with innovative production systems and the associated chains.

One NGO monitoring and evaluation officer pointed out that:

*“Illiteracy is the most significant challenge that we are facing with some local communities and potential clients, among them there are some who do not know what the long-term intentions of crop-livestock integration schemes are all about. They assume that the initiatives are put in place to completely eliminate self-funding and livestock deaths from diseases in the very short term. This compromises the uptake of climate change management options which can borrow from existing traditional knowledge*.”

From what was highlighted by the stakeholders during the study, there are asymmetries in accessing certain stakeholders, for example, farmers accessing the developers of crop-livestock integration scheme facilities due to proximity challenges. Mkwambisi [16] concurs with this viewpoint in a study done in Malawi.

#### 3.3.4 Limitations for some stakeholders on the platforms to extract real value

Any innovation platform’s roles are to provide long-term solutions to the challenges faced by especially the smaller stakeholders who are usually marginalized from core decision-making systems [21]. If not well infused and integrated, they always lag behind the innovation advancement revolutions and extract limited value from these platforms.

An extension officer indicated that:

*“The most significant challenge with the farmers is lack of capital to purchase high technology and modern equipment to use for the management of the crop-livestock integrated scheme programs, hence resulting in poor productivity and limited benefits. In response, farmers adhere to their traditional practices which do not blend and commercialize crops and livestock especially the traditional grains which have remained absent in most land allocation decisions and markets*.”

This, according to Bhatasara [19], brings about the major problem of stakeholder dissatisfaction and low usage rates with the available platforms that foster the implementation of crop-livestock integration development interventions due to reliability challenges.

## 4 Conclusion and recommendations

The study examined the knowledge, attitudes, and practices that have affected the uptake of commercially inclined crop-livestock integration as a climate change management strategy in semi-arid areas. We conclude that there are limited options for blending crop-livestock integration activities. Only three dominant options of crop-livestock integration options, namely conventional crop-livestock (63%), mixed crops-livestock (25%), and traditional grains-livestock (12%), are practiced in the study area. There is a low presence of stakeholders on the platforms regardless of the farmers’ knowledge, positive attitude, and perceptions about the potential of these blended strategies in managing climate change-related welfare deteriorating patterns. This is driven by numerous production and marketing-related constraints, including an unbalanced policy environment, inadequate financing options, and uncompetitive marketing channels. Going forward, we recommend that the traditional grains-livestock alternative be supported in the semi-arid environments as a gateway out of persistent food, income, and nutrition insecurity compounded by climate change. This can be attained by fostering public-private partnerships which can finance and support value addition systems in the production localities. This should then increase the market size, especially for the currently marginalized traditional grains-livestock products. It is our hope that this will unlock its commercialization prospects while opening up more strategic value chain nodes. In conclusion, we anticipate seeing more stakeholders on the climate change management platforms driven by crop-livestock integrated mechanisms.
